# An evaluation of the Ames Seralyzer

**DOI:** 10.1155/S1463924683000085

**Published:** 1983

**Authors:** P. M. S. Clark, P. M. G. Broughton

**Affiliations:** Department of Clinical Chemistry Wolfson Research Laboratories Queen Elizabeth Medical Centre Birmingham B15 2TH UK


Journal of Automatic Chemistry, Volume 5, Number 1 (January–March 1983), pages 22–26

### An evaluation of the Ames Seralyzer

**P. M. S. Clark and P. M. G. Broughton**

*Department of Clinical Chemistry, Wolfson Research Laboratories, Queen Elizabeth Medical Centre, Birmingham B15 2TH, UK*

### Introduction

The Ames Seralyzer Reflectance Photometer (its UK price in January 1982 was £2250 plus Value-Added Tax) employs dry reagent strips and reflectance photometry for the measurement of several analytes in serum. Reagent strips are currently available in the UK for the determination of glucose (kinetically using glucose oxidase with oxidized 3,3′, 5,5′-tetramethylbenzidine as the indicator), urea (reaction with o-phthalaldehyde which, in the acidic environment of a cation exchanger, yields a blue chromogen) and urate (uricase method yielding a coloured complex from 3-methyl-2-benzothiazolinone hydrazone and primaquine diphosphate). Other tests are being developed [1 and 2].

During analysis, light from a xenon flashtube illuminates an integrating sphere in a series of flashes. Light within the sphere strikes all surfaces. The light reflected vertically from the reagent area passes through a collimator and then through a Test Module filter to a sample detector. Other reflected light in the sphere passes through the Test Module filter to a reference detector. The reference and sample detector signals are converted by a microcomputer to a ratio; the series of reflectance measurements made during the test period is used to compute the result from the stored calibration line, and is shown on a display.

The instrument is neat and compact in appearance (height 140 mm, width 280 mm, depth 380 mm, weight 10 kg). After allowing 20 min to warm up, the appropriate Test Module is inserted, the sphere rehydrated (according to the manufacturer's instructions) and the instrument is calibrated for the specified test by entering the value for the Low Calibrator via the keyboard. A reagent strip is placed on the Reagent Table and 30 $\mu$l of Low Calibrator (diluted one in three with distilled water) is pipetted onto the centre of the reagent area. The 'Start' key is pressed immediately, and the Reagent Table pushed in. When the analysis is complete, a buzzer sounds, the 'Analyze' light goes out and the entered calibrator value is displayed. This sequence is repeated for the High Calibrator. An Error Code will indicate if calibration is unsuccessful. A similar procedure is followed for specimen analysis: all sera are diluted one in three and 30 $\mu$l is applied to the reagent strip, the Start key pressed and the Reagent Table pushed in; the result is displayed when the buzzer sounds. Over-range specimens are analysed by preparing a further three-fold dilution and pressing the 'Dil' key before the analysis. Any error or instrument malfunction is communicated via Error Codes on the display.

At the time of the evaluation, Ames stated that the Seralyzer could only be used for the analysis of serum, and that specimens for glucose analysis should not be collected in fluoride as this would inhibit the reaction in the reagent strip.

### The evaluation

The instrument was borrowed from the manufacturer (Ames Division, Miles Laboratories Ltd, Stoke Court, Stoke Poges, Slough SL2 1LY, UK) for the evaluation and was installed by their representative; Ames also provided all the necessary equipment and reagents for its operation.

### Methods

### *Precision*

Three lyophilized quality-control sera with high, medium and low analyte levels were reconstituted as directed by the manufacturer. Three fresh human serum pools were collected with high, medium and low concentrations of each analyte. Aliquots of all these sera were analysed 20 times within a single analytical run (i.e. using a single bottle of the appropriate reagent strips and within a single calibration period). Aliquots were also stored at −20°C and one of each analysed on 16–20 consecutive working days.

### *Linearity*

Specimens with high and low analyte concentrations were mixed in varying proportions to yield specimens with levels covering the full analytical range for each test. After dilution, each serum was analysed in duplicate, and further dilutions were made as necessary.

### *Effect of dilution*

Three sera with high concentrations of each analyte were analysed on a Technicon SMA 12/60. Each was then diluted with distilled water and analysed on the Seralyzer, further dilutions of over-range specimens being made as necessary.

### *Accuracy*

Nine lyophilized sera, which had been circulated through the United Kingdom National External Quality Assessment Scheme (UKNEQAS), were analysed on the Seralyzer and the results for each analyte compared with the mean value for the appropriate manual method.

### *Comparison with routine methods*

Approximately 100 specimens of serum submitted for routine analysis were analysed on the Seralyzer and on an SMA 12/60, which used the following methods: glucose-glucose oxidase; urea-diacetyl monoxime; and urate-phosphotungstate [3, 4 and 5].

### *Interferences*

The effects of haemolysed erythrocytes on urea analysis, and of ascorbic acid on urate and glucose analysis, were assessed by the addition of each, at two concentrations, to three samples of serum.

### Results and discussion

### *Precision*

The results of within- and between-batch precision studies are given in tables 1 and 2. These show that the coefficient of variation (CV) for glucose ranges from 1·5–7·2% (mean 3·5%), for urea from 4·3–12·2% (mean 6·4%), and for urate from 2·6–14·3% (mean 5·9%). These figures may be compared with recommended analytical CVs of 3·0%, 6·0% and 4·1% respectively [6]

| Analyte | | Within-batch Low | Within-batch Medium | Within-batch High | Between-batch Low | Between-batch Medium | Between-batch High |
|---|---|---|---|---|---|---|---|
| Glucose (mmol/l) | Mean | 3·9 | 5·8 | 11·6 | 3·6 | 5·8 | 13·6 |
| | SD | 0·06 | 0·09 | 0·35 | 0·18 | 0·20 | 0·74 |
| | CV% | 1·5 | 1·5 | 3·0 | 4·8 | 3·4 | 5·5 |
| | $N$ | 20·0 | 20·0 | 20·0 | 20·0 | 20·0 | 20·0 |
| Urea (mmol/l) | Mean | 2·4* | 6·1 | 19·1 | 3·0 | 6·3 | 19·2 |
| | SD | 0·15 | 0·28 | 1·23 | 0·20 | 0·29 | 1·34 |
| | CV% | 6·1 | 4·6 | 6·5 | 6·8 | 4·7 | 7·0 |
| | $N$ | 20·0 | 20·0 | 20·0 | 20·0 | 20·0 | 20·0 |
| Urate (μmol/l) | Mean | 132·0 | 308·0 | 473·0 | 105·0 | 303·0 | 505·0 |
| | SD | 6·7 | 7·9 | 37·8 | 15·1 | 14·6 | 39·3 |
| | CV% | 5·1 | 2·6 | 8·0 | 14·3 | 4·8 | 7·8 |
| | $N$ | 20·0 | 20·0 | 20·0 | 17·0 | 17·0 | 17·0 |

*These results include three values below the analytical range of the instrument and have been taken as 2·1 mmol/l. When recalculated excluding these results: mean = 2·5, SD = 0·12 mmol/l and CV = 5·0%.
Where SD = standard deviation; CV = coefficient of variation; and $N$ = number of replicate analyses.

*Table 1. Precision results for fresh human serum.*

| Analyte | | Within-batch Pathonorm L | Within-batch Equitrol | Within-batch Ortho-Abnormal | Between-batch Pathonorm L | Between-batch Equitrol | Between-batch Ortho-Abnormal |
|---|---|---|---|---|---|---|---|
| Glucose (mmol/l) | Mean | 2·5 | 5·8 | 15·4 | 2·2* | 6·4 | 15·2 |
| | SD | 0·05 | 0·17 | 0·38 | 0·16 | 0·19 | 0·76 |
| | CV% | 2·0 | 3·0 | 2·5 | 7·2 | 3·0 | 5·0 |
| | $N$ | 20·0 | 20·0 | 20·0 | 20·0 | 20·0 | 20·0 |
| Urea (mmol/l) | Mean | 3·5 | 8·0 | 19·5 | 3·7 | 9·1 | 19·3 |
| | SD | 0·43 | 0·34 | 0·90 | 0·26 | 0·56 | 1·28 |
| | CV% | 12·2 | 4·3 | 4·6 | 7·0 | 6·1 | 6·7 |
| | $N$ | 20·0 | 20·0 | 20·0 | 20·0 | 20·0 | 20·0 |
| Urate (μmol/l) | Mean | 201·0 | 346·0 | 551·0 | 204·0 | 370·0 | 574·0 |
| | SD | 12·6 | 15·0 | 19·0 | 8·5 | 18·4 | 29·2 |
| | CV% | 6·3 | 4·3 | 3·4 | 4·2 | 5·0 | 5·1 |
| | $N$ | 20·0 | 20·0 | 20·0 | 16·0 | 16·0 | 16·0 |

*These results include two values below the analytical range of the instrument and have been taken as 1·9 mmol/l. When recalculated excluding those results: mean = 2·2, SD = 0·13 mmol/l and CV = 6·0%.
(Pathonorm L from BDH, Poole, Dorset, UK; Equitrol from Tissue Culture Services, Slough, Berkshire, OrthoAbnormal from Ortho Diagnostics.)

*Table 2. Precision results for quality-control sera.*

and they are not as good as those obtained by other workers [7]. It was concluded that the precision of the Seralyzer was adequate for some clinical purposes. There was little difference between the CVs found with fresh human and quality-control sera. Two high CVs were obtained (urea [see table 2], and urate [see table 1]), but none of the results for these two experiments were outside the limits of the mean ± 3SD. When recalculated, excluding values outside the limits mean ± 2SD (one result for each), the CVs were 10·8% for urea and 12·1% for urate, indicating poor precision with these specimens.

### *Linearity*

Polynomial regression analysis of the whole data for all analytes indicated both a significant linear component and a significant non-linear component. When over-range samples requiring an additional dilution step were excluded, the linear component remained but no significant non-linearity was found. This is illustrated for glucose in figure 1; similar graphs were obtained for urea and urate. It was concluded that a linear response is found over the analytical range for all analytes, but some non-linearity is introduced by dilution of over-range specimens according to the manufacturer's recommended procedures.

### *Effect of dilution*

Polynomial regression showed that for glucose there was a significant non-linear component with over-range samples requiring further dilution before analysis on the Seralyzer (figure 2). Urea shows a similar significant non-linear component with over-range samples, but urate did not. Considering the results of the linearity and dilution experiments together, it was concluded that there is a linear relationship for all three analytes over the analytical ranges quoted by the manufacturer, but some non-linearity is introduced when further dilution is made as recommended by the manufacturer. The fact that dilution of the original specimen does not introduce non-linearity (as shown by the linear response in the first part of the graph shown in figure 2) suggests that the error may be introduced by the instrumental correction for the additional dilution step rather than by any changes in the specimen matrix.

### *Accuracy with control sera*

The results given in table 3 show that for the materials studied the Seralyzer gives results that for glucose are lower and for urea and urate determinations are higher than those of comparable

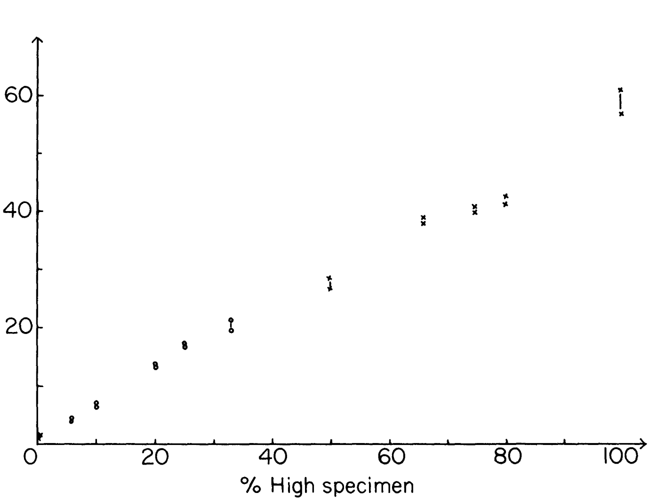

*Figure 1. Linearity of glucose determinations (x = specimens below or above [i.e. diluted] analytical range).*

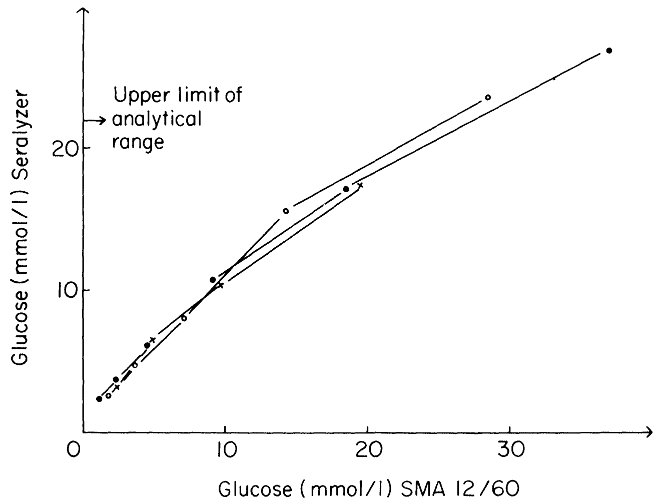

*Figure 2. Effect of dilution of specimen on glucose determinations (○ = sample 1, ● = sample 2, × = sample 3).*

*Table 3. Analysis of quality-control sera.*

| | Glucose (mmol/l) | | | Urea (mmol/l) | | | Urate (mmol/l) | | |
|---|---|---|---|---|---|---|---|---|---|
| | Manual method (glucose oxidase) | | Seralyzer | Manual method (urease) | | Seralyzer | Manual method (uricase) | | Seralyzer |
| Control material | Mean | | Mean $N=3$ | Mean | | Mean $N=3$ | Mean | | Mean $N=3$ |
| Armtrol 489 | 4·70 | (62) | 4·33 | — | — | — | 289 | (61) | 336 |
| Ortho Abnormal W24K02B | 13·67 | (62) | 11·20 | 13·91 | (62) | 15·13 | 525 | (63) | 547 |
| Ortho Abnormal 95317 | 16·99 | (66) | 15·53 | — | — | — | — | — | — |
| Pathonorm L18 | 2·20 | (67) | 2·17 | 3·66 | (68) | 4·00 | 196 | (55) | 221 |
| HIQC/4 | 8·35 | (57) | 8·10 | 7·35 | (63) | 7·60 | 335 | (63) | 351 |
| Ortho Normal W27X02B | 5·04 | (59) | 4·53 | 5·64 | (62) | 6·33 | 236 | (63) | 240 |
| Liberton L4/80 | 5·40 | (59) | 4·97 | 4·57 | (58) | 4·40 | 252 | (62) | 276 |
| Monitrol IIX SPXP9613 | — | — | — | 16·57 | (65) | 19·10 | — | — | — |
| Armtrol 551 | 4·51 | (63) | 3·93 | 8·87 | (61) | 9·70 | 300 | (67) | 326 |
| Wellcomtrol Two K9122 | 10·57 | (62) | 10·30 | 27·66 | (62) | 29·20 | 492 | (72) | 554 |
| HIQC/5 | 7·32 | (63) | 6·93 | 6·63 | (64) | 6·33 | 235 | (73) | 236 |
| Average difference % | | | −8·1 | | | +7·2 | | | +8·8 |

Manual method means were calculated through UKNEQAS, the figures in parentheses being the number of results used to calculate the mean. The manufacturer's assigned values were given for the following: Pathonorm L = glucose = 2·2 mmol/l; urea = 3·5 mmol/l; urate = 200 μmol/l: HIQC/4 = glucose = 8·35 mmol/l; urea = 7·35 mmol/l; urate = 335 μmol/l: HIQC/5 = glucose = 7·25 mmol/l; urea = 6·57 mmol/l; urate = 233 μmol/l.

manual methods. Similar results were obtained for urea and urate, but not glucose, by Thomas and co-workers [7]. There were no noticeable differences in behaviour between the different control sera (i.e. no species differences), although one lot of OrthoAbnormal (W24KO2B) showed large differences between the Seralyzer and manual glucose methods, and the number of materials studied was small. However, another lot of this material showed a smaller difference, close to the average.

### *Comparisons with routine methods*

Graphs showing the comparison between the Seralyzer results and those obtained by the SMA 12/60 are given in figures 3, 4 and 5. These show that, whilst there is a good correlation between results found with the two methods for all analytes, the Seralyzer gives statistically significantly lower results than the SMA 12/60 methods. For urate and urea, these differences are unlikely to be clinically significant, but with glucose the differences are more important. In addition, the scatter of results becomes noticeable with over-range specimens requiring further dilution (i.e. glucose: > 22·2 mmol/l; urea > 21 mmol/l; urate > 600 μmol/l).

### *Interferences*

The results are shown in table 4. Ascorbic acid was found to interfere significantly with both glucose and urate analyses. These findings confirm results quoted by the manufacturer.

No details of the effect of haemoglobin on urea analysis are given in Ames's instructions, although the manufacturer stated verbally that it did not interfere. The effect was found to be variable, with a significant increase at the lower haemoglobin level.

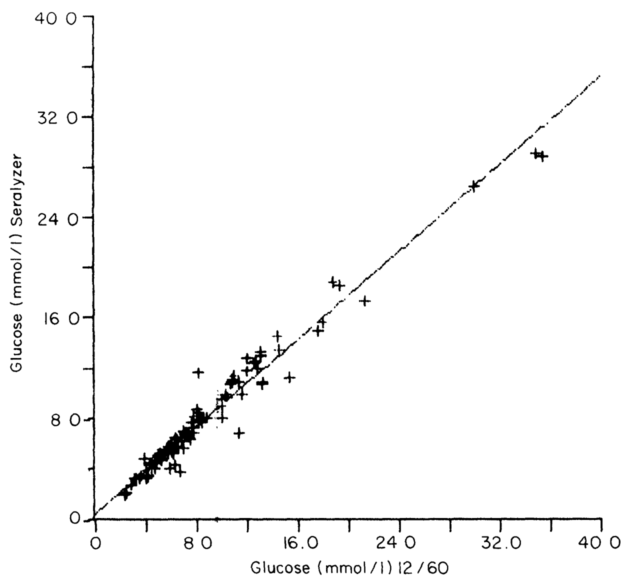

*Figure 3. Comparison of Seralyzer glucose results with SMA 12/60 results using human sera (correlation coefficient = 0·98, N = 100, y = 0·86x + 0·49).*

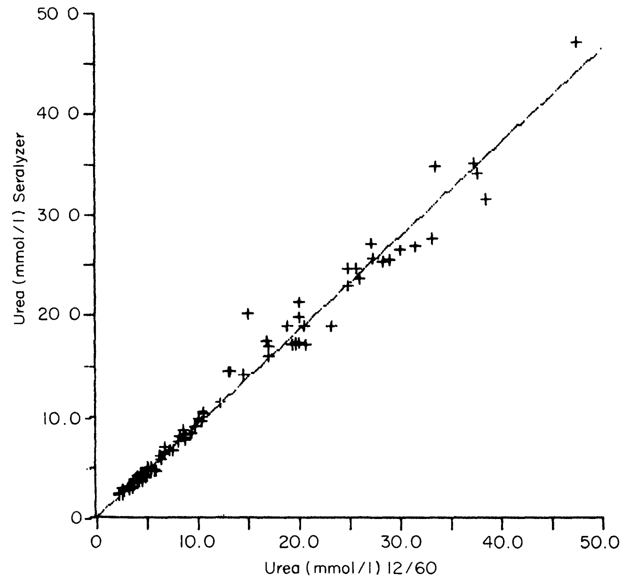

*Figure 4. Comparison of Seralyzer urea results with SMA 12/60 results using human sera (correlation coefficient = 0·99, N = 94, y = 0·93x + 0·08).*

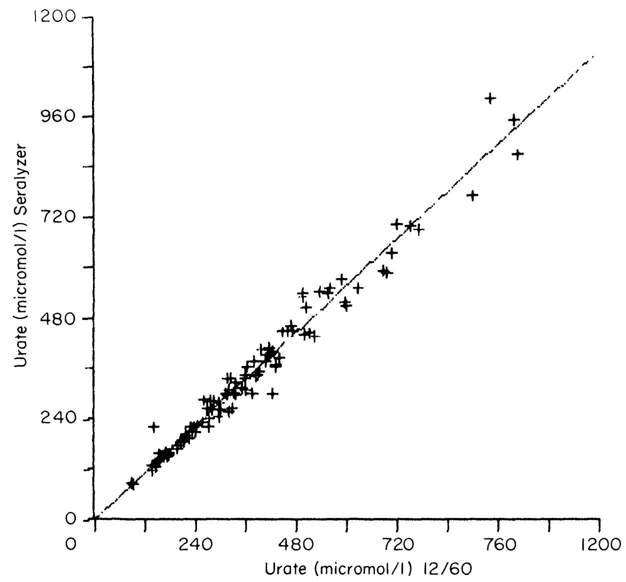

*Figure 5. Comparison of Seralyzer urate results with SMA 12/60 results using human sera (correlation coefficient = 0·99, N = 99, y = 0·93x − 2·83).*

*Table 4. Effects of added haemoglobin and ascorbic acid on urea, glucose and urate analysis.*

| Interference studied | Level of interfering substance | Sample | Percentage change | |
|---|---|---|---|---|
| Haemoglobin on urea | 3·8 g/l | 1 | +8 | |
| | | 2 | −1 | NS |
| | | 3 | +17 | |
| | 1·9 g/l | 1 | +10 | |
| | | 2 | +6 | $p < 0·01$ |
| | | 3 | +13 | |
| Ascorbic acid on glucose | 50 mg/l | 1 | −13 | |
| | | 2 | −14 | $p < 0·001$ |
| | | 3 | −6 | |
| | 25 mg/l | 1 | −8 | |
| | | 2 | −11 | $p < 0·01$ |
| | | 3 | −3 | |
| Ascorbic acid on urate | 100 mg/l | 1 | −35 | |
| | | 2 | −39 | $p < 0·001$ |
| | | 3 | −37 | |
| | 50 mg/l | 1 | −29 | |
| | | 2 | −32 | $p < 0·001$ |
| | | 3 | −34 | |

### *Dependability*

During the 18-week period of the evaluation, no major faults developed and relatively few Error Codes occurred. During the evaluation, recalibration (other than that performed daily, for new bottles of strips, or when switched on or power cut-off) was necessary when the reanalysis of calibrators and controls indicated that either calibrator or control values were outside the manufacturer's limits. This occurred 10 times for glucose, 10 times for urea and four times for urate. With glucose and urate this was because the results for Omega controls fell outside the limits suggested by Ames. No analytical reason could be found for this, and inspection of the control graphs showed that urate results were always high and glucose results low. No explanation of this was given by the manufacturer, and subsequently recalibration was only carried out if the control values were well outside these limits.

### *Instructions*

These consist of package inserts, enclosed with each bottle of reagent strips and each set of calibrators, and an operating manual. They give a good account of the theoretical principles of the instrument and the chemical reactions used, but in order to perform the three tests evaluated here, seven items of literature must be consulted. This is highly repetitive and often confusing, particularly when some data are given in mg/dl and others in SI units. Package inserts recommend hydration of the integrating sphere every 8 h for glucose, and every 15 min for urea, but this is not mentioned for urate. Although each calibration by the analysis of calibrators and controls was checked, as recommended by the manufacturer's representative, this is not mentioned in any of the printed instructions, and quality control receives scant attention or explanation.

### *Electrical and mechanical construction*

Our engineering staff examined the machine without making rigorous mechanical and electrical tests, and found the Seralyzer to be basically safe. However, the Reagent Table is easily contaminated and difficult to clean. Contamination with serum spilling from the reagent strips, particularly the urea strips, was frequent—even with an experienced operator. A gap between the upper and lower cases of the instrument might also allow some specimen spillage into the instrument.

The colour and texture of the case of the instrument made contamination difficult to see and so cleaning was difficult. The keyboard was not easily decontaminated, and a membrane-type keyboard would therefore be preferable.

### *Running costs*

The cost of consumables, together with direct labour times [8], for glucose, urea and urate are given in table 5. This shows that calibration costs are relatively high with small work-loads, but the cost per test decreases rapidly for larger work-loads. However, the operator will then soon become bored, and his results deteriorate with more than about 50 tests per day. To obviate this by employing a second operator would further increase costs.

*Table 5. Cost of consumables (including Value-Added Tax) and direct labour time per test for different daily work-loads.*

| | Glucose | | Urea | | Urate | |
|---|---|---|---|---|---|---|
| | £ | min | £ | min | £ | min |
| *Calibration sequence only* | | | | | | |
| Consumables | 2·04 | | 2·04 | | 2·52 | |
| Labour | | 16 | | 14 | | 19 |
| *Single test only* | | | | | | |
| Consumables | 0·23 | | 0·23 | | 0·31 | |
| Labour | | 2 | | 3 | | 4 |
| *Tests per day* | | | | | | |
| 1 Consumables | 2·27 | | 2·27 | | 2·83 | |
| Labour | | 18 | | 17 | | 23 |
| 5 Consumables | 0·63 | | 0·63 | | 0·81 | |
| Labour | | 5·2 | | 5·8 | | 7·8 |
| 20 Consumables | 0·33 | | 0·33 | | 0·44 | |
| Labour | | 2·8 | | 3·7 | | 5·0 |

### Conclusions

The Seralyzer is an inexpensive instrument, which is quick and simple to use, and its performance in measuring serum glucose, urea and urate is adequate for some clinical purposes. However, it does have several major disadvantages. It is restricted to the analysis of serum and cannot measure low glucose levels. All specimens need predilution and the instrument was found to be labour-intensive and operator-dependent.

Since the Seralyzer may be used outside the hospital laboratory, its accuracy is of some concern—particularly with glucose. With quality-control sera, results for all three analytes differ considerably from those found with comparable manual methods. This restricts the ability of the operator to check the accuracy of his results with commercial quality-control sera. If he uses materials with assigned values obtained with Seralyzer methods, he is merely checking one Seralyzer against another. The user would be unable to check performance of the instrument with external quality-assessment schemes, which at present do not quote method-mean values for the Ames Seralyzer. These factors are particularly important if the instrument is used by staff who are not familiar with quality-control procedures and, in general, this subject is given inadequate attention in the manufacturer's literature.

Although the range of tests possible on the Seralyzer may be extended in the future, the present limitation of the instrument to glucose, urea and urate measurements is unlikely to make it useful in hospital laboratories in the UK, particularly as there are cheaper alternatives available for blood glucose measurements [9]. It might, however, find an application where few samples need to be analysed quickly, although it must be emphasized that this will require some skill, dexterity and acumen.

### Acknowledgements

The authors wish to thank the Ames Division of Miles for the loan of the instrument and for their co-operation during the evaluation. The financial support of the DHSS is gratefully acknowledged.

### References

1. Greyson, J., *Journal of Automatic Chemistry*, **3** (1981), 66.
2. Zipp, A., *Journal of Automatic Chemistry*, **3** (1981), 71.
3. Trinder, P., *Annals of Clinical Biochemistry*, **6** (1969), 24.
4. March, W. M., Fingerhut, B. and Miller, H., *Clinical Chemistry*, **11** (1965), 624.
5. Musser, A. W. and Ortigoza, C., *Technical Bulletin of Registered Medical Technicians*, **36** (1969), 2125.
6. Statland, B. E., *Clinical Biochemistry Reviews*, **3** (1982), 1.
7. Thomas, L., Plischke, W. and Storz, G., *Annals of Clinical Biochemistry*, **19** (1982), 214.
8. Broughton, P. M. G. and Hogan, T. C., *Annals of Clinical Biochemistry*, **18** (1981), 330.
9. Webb, D. J., Lovesay, J. M., Ellis, A. and Knight, A. H., *British Medical Journal*, **280** (1980), 362.

